# Early Symptomatic Syndromes Eliciting Neurodevelopmental Clinical Examinations

**DOI:** 10.1155/2013/710570

**Published:** 2014-01-06

**Authors:** Christopher Gillberg, Elisabeth Fernell, Helen Minnis

**Affiliations:** ^1^Gillberg Neuropsychiatry Centre, Sahlgrenska Academy, University of Gothenburg, Kungsgatan 12, 411 19 Gothenburg, Sweden; ^2^Glasgow University, UK

This special issue is devoted to the concept of ESSENCE (Early Symptomatic Syndromes Eliciting Neurodevelopmental Clinical Examinations). It is an acronym that one of us coined some years ago [1] with a view to highlighting the clinical reality of children (and their parents) presenting in first, second, or third tier clinical settings with, usually complex, impairing developmental symptoms already in early childhood. The children are reported to have problems in the fields of (a) general development, (b) communication and language, (c) social interrelatedness, (d) motor coordination, (e) attention/“listening,” (f) activity, (g) behavior, (h) mood, and/or (i) sleep. Children with major difficulties in one or more (usually several) of these fields will be seen by health visitors, nurses, social workers, education (including preschool) specialists, pediatricians, GPs, speech and language therapists, child neurologists, child psychiatrists, psychologists, neurophysiologists, dentists, clinical geneticists, occupational therapists, and physiotherapists, but, in the vast majority of cases they will be seen only by one of these specialists, when, in fact, they would have needed the input of two or more (occasionally even all) of the “experts” referred to.

Categorical diagnosis is an integral part of everyday clinical and research practice. We are so insistent on the distinction between disorder and not disorder (normalcy) that clinics and clinicians become more and more specialized and cater to the needs of children with “Autism Spectrum Disorder/ASD only,” “Attention-Deficit/Hyperactivity Disorder/ADHD only,” “Language Disorder only,” “Reactive Attachment Disorder/RAD only,” or “Tourette syndrome only.” This has led to a situation in which the typical clinical diffuseness of disorder has come to be underestimated.

At the same time, there is growing acceptance that coexistence of disorders and sharing of symptoms across disorders (so-called comorbidity, a misnomer if ever there was one, seeing as we are usually not dealing with completely separate coexisting disorders) are the rule rather than the exception (e.g., [2]). This was pointed out more than a quarter of a century ago [3], but, in clinical practice, this insight has not led to new approaches when trying to address the needs of children and families with “complex needs.” Instead, diversification has boomed.

There are legislational, scientific, and clinical attempts to separate out children with certain disorders/diagnosis; for example, ASD from those who do not meet criteria for the disorder/diagnosis. The goal is usually provide better societal guidelines, more focused attempts at finding the causes, and more specific services. Children with ADHD are targeted in similar ways, even though legislation has yet to catch up with them. The same holds for children with Language Disorder, visual impairments, and hearing deficits (children who may, or may not, have additional impairments as regard to general cognition, motor performance, ADHD, or ASD).

ASD and ADHD, long treated and, believed to be, completely separate and recognizable “disorders,” are now increasingly often diagnosed “together” within the same individual, and there is growing awareness that they sometimes overlap, constitute amalgams of problems, and that in some families they separate together and probably represent different aspects of the same underlying disorder [4].

The overlap, shared and nonshared symptoms and etiologies of these named disorders are in focus in this special issue.

There are 14 papers altogether in the volume. They deal with a variety of ESSENCE-related issues ranging from maltreatment and RAD (and the difficult issue of how to study the effects of early intervention aiming to stop maltreatment and provide safe and nurturing care), through autism from preschool to adult life (including a study showing that autism diagnosed early in life is always comorbid with other disorders, and a literature review indicating that pain sensitivity is, in fact, *not* decreased in autism), language delay (which can only be screened for by face-to-face assessment), eating problems (that are shown to be comorbid with ASD and ADHD symptomatology in a very high proportion of cases), and cerebral palsy (in which the prevalence of mental health problems, including ADHD, appears to currently be grossly underestimated) to more general mental health problems (and the use of child psychiatric and school psychology services that is associated with such problems). Methodologies covered in the special issue range from longitudinal population-based screening and clinical assessment studies, through randomized controlled trials and large-scale epidemiological twin research, to position and PRISMA-guideline systematic review papers. There is a wealth of information for clinicians and researchers alike, and it is expected that the issue on ESSENCE will prove to be a landmark in the development of new approaches to screening and intervention for children and adults affected by neurodevelopmental disorders. ESSENCE affect about one in ten individuals across the planet, and we are dealing with a huge public health problem. ESSENCE, from now on, must be given highest priority both in clinical research and practice.

*Christopher Gillberg* *Christopher Gillberg* *Elisabeth Fernell* *Elisabeth Fernell* *Helen Minnis* *Helen Minnis*
